# Possible Mechanisms of Mercury Toxicity and Cancer Promotion: Involvement of Gap Junction Intercellular Communications and Inflammatory Cytokines

**DOI:** 10.1155/2017/7028583

**Published:** 2017-12-21

**Authors:** Roberto Zefferino, Claudia Piccoli, Nunzia Ricciardi, Rosella Scrima, Nazzareno Capitanio

**Affiliations:** ^1^Department of Medical and Surgical Sciences, University of Foggia, Via L. Pinto 1, 71122 Foggia, Italy; ^2^Department of Clinical and Experimental Medicine, University of Foggia, Via L. Pinto 1, 71122 Foggia, Italy

## Abstract

A number of observations indicate that heavy metals are able to alter cellular metabolic pathways through induction of a prooxidative state. Nevertheless, the outcome of heavy metal-mediated effects in the development of human diseases is debated and needs further insights. Cancer is a well-established DNA mutation-linked disease; however, epigenetic events are perhaps more important and harmful than genetic alterations. Unfortunately, we do not have reliable screening methods to assess/validate the epigenetic (promoter) effects of a physical or a chemical agent. We propose a mechanism of action whereby mercury acts as a possible promoter carcinogen. In the present contribution, we resume our previous studies on mercury tested at concentrations comparable with its occurrence as environmental pollutant. It is shown that Hg(II) elicits a prooxidative state in keratinocytes linked to inhibition of gap junction-mediated intercellular communication and proinflammatory cytokine production. These combined effects may on one hand isolate cells from tissue-specific homeostasis promoting their proliferation and on the other hand tamper the immune system defense/surveillance checkmating the whole organism. Since Hg(II) is not a mutagenic/genotoxic compound directly affecting gene expression, in a broader sense, mercury might be an example of an epigenetic tumor promoter or, further expanding this concept, a “metagenetic” effector.

## 1. Mechanisms of the Prooxidative Activity of Mercury

The cytotoxic effect of mercury in its divalent ionic form Hg^2+^ has been linked to cellular oxidative stress by many authors [1–3]. The general belief is that given the well-known reactivity of Hg^2+^ with thiols to form mercaptans this may result in depletion of the thiol-based antioxidant buffers constituted in cells mainly by glutathione. Consistent with this notion, increased GSSG/GSH ratio and H_2_O_2_ production have been repeatedly reported in literature in different cell phenotypes exposed to mercury-containing compounds.

Accordingly, our group found that exposure of cultured human keratinocytes (HK) to nanomolar concentrations of HgCl_2_ for 24 h caused a 40% decrease of the fluorescence signal associated to the free thiol-reacting probe Alexa Fluor 594 C5 maleimide as assessed by confocal microscopy imaging [4]. Moreover, direct measurement of the reduced and oxidized glutathione resulted in a twofold increase of the relative amount of GSSG thus confirming the negative effect of Hg^2+^ on the free thiol-based antioxidant cellular pool. Consistently, when the intracellular level of reactive oxygen species (ROS) was measured by the redox-sensitive fluorescent probe DCF, a fivefold increase of the signal was detectable by confocal microscopy in Hg^2+^-treated HK as compared with untreated cells. Higher resolution of the imaged intracellular fluorescence revealed that the brighter signal was localized in the mitochondrial compartment. Similar results were attained with the superoxide anion- (O_2_^•−^) specific mitotropic probe MitoSOX. However, both measurement of the mitochondrial respiratory chain activity as well as of the mitochondrial transmembrane potential (ΔΨ_m_) (by the TMRE probe) did not show appreciable differences between untreated and Hg^2+^-treated HK. Overall, these results suggest that the HgCl_2_-mediated oxidative unbalance was likely due to depletion/impairment of the antioxidant buffering system rather than to increased ROS production at least of mitochondrial origin. However, it must be considered that the intracellular concentration of free thiol groups is estimated in the millimolar range, whereas the concentration of HgCl_2_ was 5-6 order of magnitude lower. Even considering the small volume of the cell layer of HK in culture and all the Hg^2+^ available in the medium, the amount of Hg^2+^ was still largely substoichiometric with respect to the intracellular free thiol groups. This ruled out a direct involvement of Hg^2+^ in the oxidative modification of the thiol-based redox buffering rather suggesting Hg^2+^-mediated modification of specific catalytic reactions controlling the ROS homeostasis.

**Figure 1:**
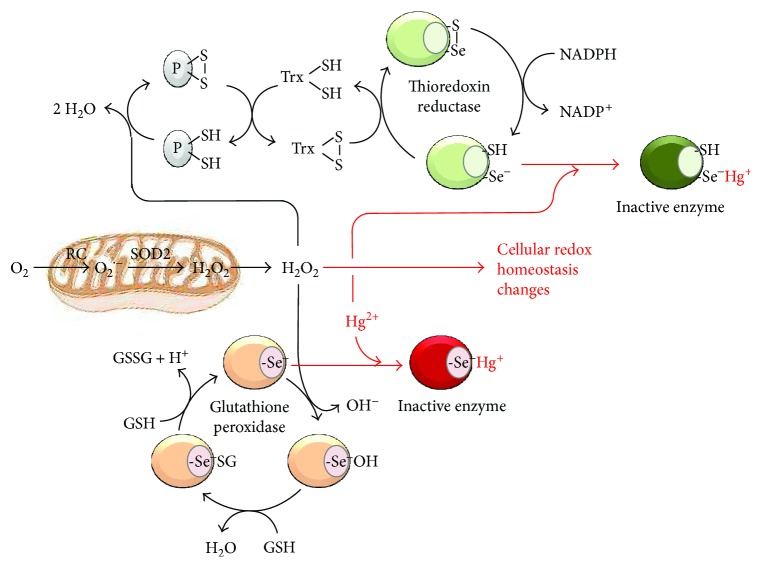
Proposed mechanism for the prooxidative action of Hg(II). Mercury ion (Hg^2+^) is shown to bind to the dissociated form of the selenol (-Se^−^) moiety of the catalytic selenocysteine residue of glutathione peroxidase and thioredoxin reductase thereby inactivating the enzymes; the result is an enhanced level of reactive oxygen species because of their lower inactivation. The catalytic cycles of the two antioxidant enzymes are also shown: glutathione peroxidase converts H_2_O_2_ in 2 H_2_O molecules at expense of 2 reduced glutathione molecules (GSH), which are oxidized to GSSG; thioredoxin reductase reduces oxidized thioredoxin (Txr) at the expense of NADPH thereby enabling reduced Txr to preserve the redox state of protein cysteines (P) from the H_2_O_2_-mediated oxidation. Mitochondria is illustrated as a major intracellular producer of ROS generated from electron leaks from the respiratory chain (RC) to O_2_ to form the superoxide anion (O_2_^•−^). This is further converted in H_2_O_2_ by the mitochondrial isoform of the superoxide dismutase (SOD_2_).

Evidences have been provided that mercury-containing compounds induce changes in the redox state of the sulphydrilic lateral chain of cysteines in the active site of several enzymes [5–8] with some of these possibly involved in the control of the balance between ROS production and ROS scavenging. Importantly, some antioxidant enzymes such as glutathione peroxidases and thioredoxin reductase contain a residue of selenocysteine in their active site [9]. The selenol group (-Se-H) exhibits a pKa about three unit lower than that of the -SH group (i.e, 5.2 versus 8.3) therefore largely existing at physiological pH in the dissociated and more nucleophilic form (-Se^−^). Consequently, the selenol group displays a much higher reactivity toward Hg^2+^ as compared with the thiol group. On this basis, we proposed that the prooxidant activity of Hg^2+^ attainable at low concentration in HK is mainly mediated by its selective reaction with seleno enzymes involved in the “detoxification” of reactive oxygen species (see Figure 1).

To support our conclusion, there are a number of studies. In 2012, Branco et al. [10] observed in Zeabra-seabreams the histopathological alterations of Hg^2+^ in the liver and kidney and correlated this effect to a reduction of thioredoxin reductase activity. Importantly, the coexposure of Hg^2+^ and Se prevented both the organ lesions and the inhibition of the thioredoxin reductase.

Although data on the mechanisms mediating Hg0 neurotoxicity are scarce, existing evidence suggests that changes in the redox state of -SH-containing proteins plays a critical role [5–8]. However, based on the high affinity of Hg^2+^ (derived from Hg0) for selenols [11], it is reasonable to suggest that selenoproteins could also mediate the neurotoxic effects observed after Hg0 exposure. Carvalho and coworkers [12] observed that the selenoprotein thioredoxin reductase (TrxR) is selectively inhibited by Hg^2+^ and concluded that the significant potency of the mercurial to bind to the selenol group in the active site of TrxR represents a major molecular mechanism of its toxicity. Because of the probable interaction between Hg^2+^ (derived from Hg0) and selenols in the CNS, the potential involvement of selenoproteins in the neurotoxicity elicited by Hg0 represents an important research field that deserves further attention. This is believed because (i) Hg^2+^ toxicity is antagonized by selenium compounds [13, 14], (ii) Hg^2+^, which is generated in the SNC after Hg0 oxidation, inhibits the activity of selenoproteins by interacting with their selenol group [12], and (iii) miners occupationally exposed to Hg0 had lower levels of plasma selenium when compared with control individuals [15].

## 2. Effect of Mecury on Gap Junction Intercellular Communication and Cytokine Release

Gap junctions play a central role in coordinating intercellular signal-transduction pathways to control tissue homeostasis. A family of transmembrane proteins, called connexins, assembles at the level of the plasma membrane to form exameric emichannels or connexons [16]; the docking of connexons of adjacent cells forms what is called gap junction. Opening of the gap junction allows the cytoplasm of the connected cells to establish a continuum whereby low molecular weight (<500 D) metabolites, messengers, and ions can freely flow following concentration gradients. The opening/closing of the gap junction “gates” is controlled by a number of posttranslational covalent modifications linked to the activation of various signaling pathways [16]. To notice, the intercellular communication has been recently recognized to occur by different modalities consisting in nanotube connections and transfer of exosomic microvesicles between cells [17, 18]. In both cases, molecules with bio-signaling properties, genetic materials (like small RNAs), and even subcellular organelles were shown to be transferrable. The mechanisms controlling non gap junction-mediated intercellular communication are, however, yet to be fully elucidated. Deregulation of gap junctional intercellular communication is a common phenotype of cancer cells, and several lines of evidence support its involvement in the carcinogenesis process. The basic principle underlying this hypothesis is that functional isolation/segregation of a cell from the neighbor cellular environment promotes autonomous cell cycling not synchronized/regulated by the tissue biochemical cues. Once this condition becomes chronic, it primes/fosters the acquisition of a transformed phenotype. Concomitant genetic alterations can ultimately lead to cancer. Accordingly, many carcinogens, like environmental heavy-metal chemical pollutants, are known to negatively modulate GJIC though the molecular mechanism is still debated.

If an enhanced level of ROS is kept below a cytotoxic threshold, they serve as bio-signals to evoke cellular adaptation. Notably, many protein kinase/phosphatases are redox sensitive as well as transcription factors thus suggesting the occurrence of an interplay among several signaling pathways. In our previous studies, we reported that prooxidant conditioning of HK exposed to low concentration of HgCl_2_ resulted in inhibition of the GJIC accompanied with altered electrophoretic migration of connexin 43 reported to be caused by phosphorylation of the protein. Importantly, cotreatment of HK with antioxidant or inhibitor of protein kinase C prevented completely the Hg^2+^-mediated blockage of the GJIC [19]. On this basis, we proposed a mechanistic model whereby exposure of HK to Hg^2+^ causes enhanced ROS production by inhibition of selenocysteine-containing antioxidant enzyme. This stimulates member of the protein kinase C family, proved to be redox sensitive, which in turn hampers/closes the GJIC by phosphorylating connexin 43. The physiological rationale of this adaptive mechanism to a prooxidative setting remains to be fully understood. However, it might be a protective mechanism evolutionary selected to limit the spread of potentially harmful species toward in-contact neighbor cells.

Recent studies have advanced our understanding that the regulation of immune responses is not only confined to immunocompetent cells. Upon stimulation, keratinocytes are capable of releasing various factors and expressing pattern recognition receptors (PRRs) that are significantly involved in the innate immune response. Indeed, in response to challenge with microbes or microbial-derived substances, the activation and nuclear translocation of NF-*κ*B and the production of nitric oxide (NO) and inflammatory cytokines occur in keratinocytes, in a TLR-dependent manner [20]. On this basis, we have investigated the impact of Hg^2+^ on the LPS-mediated immune activation of HK. We found that nanomolar concentrations of HgCl_2_ significantly reduced the release of TNF-*α* and IL-1*β* in LPS-stimulated cells and that this effect was redox sensitive as it was abrogated by antioxidant cotreatment [21]. Although the mechanism linking mercury-mediated ROS accumulation to inhibition of cytokine production remains to be detailed, nevertheless our finding supports the long known immunosuppressive role of mercury compounds on immunocompetent cells [22].

## 3. Mercury as Cancer Promoter: a Sensu Lato Epigenetic Modifier

Epigenetics is defined as a complex of events leading to the control and regulation of gene expression without the involvement of any change in the genetic sequence. The processes, which can be altered in the epigenetic modification, include DNA methylation, histone modification, RNA regulation, DNA repair, transcription, RNA stability, alternative RNA splicing, protein degradation, gene copy number, and transposon activation. Pollutants such as heavy metals, as well as pharmaceuticals, hormones, nutrition, and behavior, can all modify the expression of genes. The pattern of the epigenetic alterations can be both transient and permanent to be transmitted to offsprings.

Epigenetic effects of heavy metals have been investigated extensively, particularly arsenic, cadmium, cobalt, chromium, nickel, and mercury. Regarding arsenic, numerous authors observed global hypomethylation, but also global and gene-specific hypermethylation particularly P53, plus histone modification (alkylation) and increment of miRNAs as miR-22 or decrement of miR-210 and miR-19a [23]. For cadmium, both global hypomethylation and hypermethylation have been reported depending on the exposure time [24–26]. For cobalt, the epigenetic effects published consisted enhanced oxidative stress, proinflammatory effects, and abnormal apoptosis [27]. It has been proved that chromium caused P16 and Gpt hypermethylation, as well as histone modifications as alkylation and phosphorylation [28]. Nickel was able to induce hypermethylation of ATF-1, HIF-1 gpt, Rb, and P16, and then histone modification as alkylation and phosphorylation [28–31]. Finally, mercury induced global hypomethylation and hypermethylation of the signaling G protein GTPase Rnd2 [32, 33].

In addition to the abovementioned modifications, dynamically controlling chromatin remodeling, gene transcription can also be indirectly modulated by the availability of metabolites functioning as substrates or effectors of epigenetic modifier enzymes as well as from microRNAs (miRNAs). This emerging notion let us to reconsider epigenetics in a broader sense including all the conditions directly or indirectly leading to acute or chronic modification of gene expression. In this acceptation, the intercellular communication may be considered an epigenetic process regulating gene expression. Indeed, the low molecular weight of the 22-nucleotide noncoding miRNAs enables them to be transferred through gap junction establishing an intercellular genetic cross-talk. The intercellular genetic communication is now emerging as an essential requirement for coordination of cell proliferation and differentiation and has an important role in many cellular processes [34].

A promoter carcinogen is by definition any agent able to determinate an uncontrolled proliferation. This action, in the opinion of several authors, including ourselves, would be linked to inhibition of GJIC. In fact, cells deprived by inhibitory control of neighboring cells start to proliferate. This aspect is, obviously, only one part of the complex and multistep carcinogenic process where microenvironmental factors play conditioning activities. In this context, the cytokine network might contribute to determine the destiny of cell towards the death or the survival. Accordingly, numerous observations proved a link between inflammation and cancer. If a substance apart from the inhibition of GJIC is also able to reduce the release of proinflammatory cytokines, its carcinogenic potency would result incremented.

**Figure 2:**
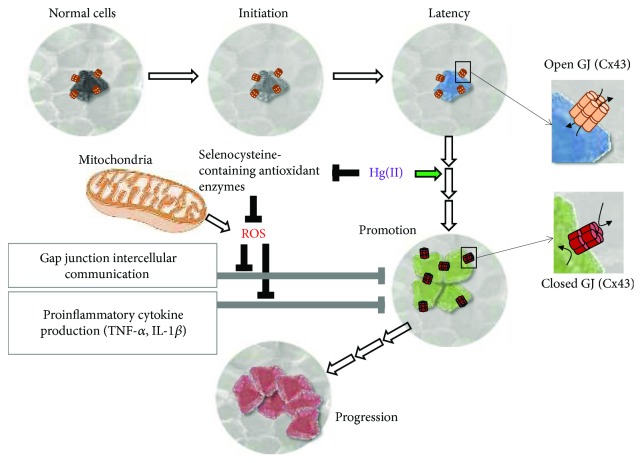
Proposed role of mercury as epigenetic/promoter carcinogen. The progressive multistep transformation of a normal cell to a cancer cell is shown schematically. The indicated phases (i.e., initiation → latency → promotion → progression) are those commonly accepted for cancer development. Mercury (Hg(II)) is indicated to act in the promotion phase by causing an unbalance in the reactive oxygen species (ROS) homeostasis accomplished by selective inhibition of selenocysteine antioxidant enzymes. Mitochondria are also shown as a major ROS generator. The Hg(II)-induced prooxidative state in turn would result in inhibition of the gap junction intercellular communication (GJIC) and of the proinflammatory cytokine release. Both mechanisms might on one hand isolate cells from tissue-specific homeostasis promoting their proliferation and on the other hand tamper the immune system defense/surveillance checkmating the whole organism. The Cx43-related open gap junction is shown as progressively closing in the transitions following the “latent” state.

In keeping the above defined concepts and on the basis of the evidences provided by our experimental work on HK, as well as by others, we hypothesize that mercury might be an epigenetic carcinogen. Indeed, Hg(II) fulfils both the capacity to induce an inhibition of the GJIC and that to induce immunosuppressive effects. This double mechanism of toxicity by Hg(II) might overcome the organism defences that exploit inflammation to contrast the cell hyperproliferation. Both these Hg-mediated outcomes are linked to an upstream alteration of the intracellular redox tone likely caused by a specific inhibition of antioxidant enzymes containing selenocysteines (see scheme of Figure 2).

The “carcinogen as mutagen” has become a “default” paradigm. This is partly because the carcinogenicity of a compound is commonly assessed by the so-called “genotoxicity” in vitro assays and tests to evaluate whether epigenetic toxicology are lacking in the routine. Consequently, mercury has been classified in group 3 by IARC (International Agency Research Cancer) which means “not classifiable as to their carcinogenicity to humans” [35]. However, if a toxic compound inhibits the intercellular communication, it might potentially act as a cancer “promoter,” although resulting nongenotoxic. In the case of the GJIC, the impact on it of chemical agent can be easily assessed as a number of protocols have been developed [36, 37]. Most notably, for some of these protocols measuring intercellular fluorescent dye transfer, either a specialized expertise or particular instrumentations, is required in making the setup of the assay easily accessible to every basic laboratory.

All together, the abovementioned considerations let us to conclude that *there is something more beyond the epigenetic effect*. Alyea et al. [38] stated that epigenetic effects caused by metals and toxic compounds always appear at low concentration, even below what they called no-observed-adverse-effect level (NOAEL), and then the authors concluded that the epigenome dynamic variability is not completely characterized. Thus, given the state of our current scientific knowledge, an epigenetic change cannot be contextualized as adverse in the absence of a phenotypic anchor. They concluded that more research is needed in this area to perform additional epigenetic studies that include apical end points with full-dose response curves, in order to gain a more comprehensive understanding of adverse health outcomes that could be causally linked to epigenetic changes. These conclusions are useful to approach in a new and unexpected way the epigenetic effect, trying to evaluate more critically where this event drives. It is known that cells, tissues, organs, apparatuses, and organisms are able to develop feedback mechanisms that counteract modifications induced by toxic substances. Hence, though it is useful to investigate canonical epigenetic modifications, it will be even more useful in the future to ask ourselves what links these epigenetic effects with other epigenetic effects that could be defined “beyond/after epigenetic effect” to differentiate them. Several authors as Alyea et al. [38] consider inhibition of intercellular communication, enhanced oxidative stress, proinflammatory effects, and abnormal apoptosis/survival as “bona fide” epigenetic effects. They could be also defined, in our opinion, in a single word as “metagenetic effects” using an ancient Greek prefix that means beyond.

## Abbreviations

GJIC: Gap junction intercellular communication
PKC: Protein kinase C
PKA: Protein kinase A
LPS: Lipopolysaccharide—endotoxin
IL-1*β*: Interleukin 1 beta
TNF-*α:*: Tumor necrosis factor alpha
NOAEL: No-observed-adverse-effect level.

## Data Access

The authors are available to share their data.
